# Depressive disorders in patients with sleep apnea syndrome

**DOI:** 10.1192/j.eurpsy.2023.1260

**Published:** 2023-07-19

**Authors:** N. Rmadi, N. Kammoun, N. Kotti, R. Masmoudi, S. Rekik, M. L. Masmoudi, K. Jmal Hammami, J. Masmoudi, S. Kammoun, S. Msaed, M. Hajjaji

**Affiliations:** 1 Department Of Occupational Medicine, HEDI CHAKER hospital; 2 Tunisian Ocupational Health and Safety Institute; 3 Psychiatry A Department; 4 Pneumology department, HEDI CHAKER hospital, SFAX, Tunisia

## Abstract

**Introduction:**

A growing body of literature has documented that obstructive sleep apnea syndrome (OSAS) is associated with an increased risk of depressive disorders.

**Objectives:**

This study aimed to determine whether depression is associated with OSAS.

**Methods:**

This was a descriptive prospective comparative study conducted over two years among patients with OSAS. Excessive daytime sleepiness was assessed by the Epworth Sleepiness Score (SES). The presence and intensity of depressive symptoms were screened using the Patient Health Questionnaire PHQ9. Data were analysed using SPSS software.

**Results:**

A total of 139 patients participated in the survey with an average age of 48.98 ± 9.80 years. According to the SES, the study population was divided into two groups: a group including 70 subjects with normal SES (< 11); and a group including 69 subjects with pathological SES (≥11). The PHQ9 depression score was higher in sleepy subjects with SES ≥ 11 compared to non-sleepless subjects; the difference being very highly significant (PHQ9=11.97 ± 4.99 and 6.54 ± 5.27 respectively; p=0.0000). The frequency of mild to moderate depressive disorders was found to be greater in non-sleepy subjects (94.3% and 78.3% respectively; p=0.007). For moderately severe to severe depression, their frequency was more marked in sleepy subjects (21.7% and 5.7% respectively; p=0.007).

**Conclusions:**

Depressive disorders constitute a major comorbidity in OSAS. Therefore, it is necessary to improve the quality of these patients’ health by the early detection of the symptoms of overlapping OSAS and depression.

**Disclosure of Interest:**

None Declared

